# Examining the effects of organizational influencers on the implementation of clinical innovations: A qualitative analysis

**DOI:** 10.1017/ash.2023.274

**Published:** 2023-09-29

**Authors:** Demetrius Solomon, Vishala Parmasad, Douglas Wiegmann, Jukrin Moon, Lucas Schulz, Alexander Lepak, Aurora Pop-Vicas, Ryan Ferren, John OHoro, Nicholas Bennett, Alec Fitzsimmons, Nasia Safdar, Sara Hernandez

## Abstract

**Background:** The FIRST Trial is a 5-year study funded by the Agency for Healthcare Research and Quality. Our investigation is situated within a more extensive study to restrict fluoroquinolone antibiotics by requiring providers to obtain authorization from an infectious disease physician before prescribing fluoroquinolones. Our research team is performing a systematic evaluation to identify organizational characteristics and influencers of the fluoroquinolone preprescription authorization implementation process to understand variables that may facilitate or hinder implementation success. **Methods:** To address this critical gap, we present a qualitative analysis from our ongoing, multisite research project aimed at systematically assessing the adoption of an antimicrobial stewardship intervention in the form of an EHR-integrated best-practice alert (BPA) at each site to identify work system factors that impact uptake and variability in the implementation of the BPA at each location. The evaluation provides a detailed explanation of activities through the implementation process (eg, before implementation, during implementation, and after implementation) to assess how an organization effectively negotiates the phases and transitions, ultimately influencing the impact of the intervention. We have used a contextual determinant framework (CFIR) that has enabled us to perform a systematic and comprehensive exploration and identification of potential explanatory themes or variables to shed light on the complex social phenomenon of implementation. **Results:** Participants who will be a part of our poster presentation will learn about implementing a BPA, the potential barriers to implementation, and strategies for overcoming these barriers. Stakeholders within our study include site coordinators, medical doctors, nurses, pharmacists, and clinical informaticists. Our analysis synthesizes their experiences implementing and sustaining this evidence-based antimicrobial stewardship intervention. It includes (1) a detailed description of the process of change, (2) work-system factors (eg, inner setting and outer setting) that they believe influenced the success of the intervention, (3) barriers and facilitators (eg, CFIR constructs) within the implementation process; and (4) description of how these could have influenced the outcomes of interest (eg, implementation and intervention effectiveness). **Conclusions:** Our research is expected to advance patient safety research and initiatives by providing a more robust approach to performing systematic intervention evaluations. By outlining stakeholders’ experiences within our study, implementation leaders within healthcare systems will utilize our findings to aid them in their design and implementation process when designing and implementing similar types of healthcare interventions.

**Disclosures:** None

